# Supplementary material for: Metagenomic and metabolomic analysis of a bryozoan-associated microbial complex from the Hanjiang River, China
Source: PLoS One. 2026 Nov 2;21(11):e0358304. doi: 10.1371/journal.pone.0358304 (PMC13726963; doi:10.1371/journal.pone.0358304)
Supplement: Supplementary file 1 [file pone.0358304.s001.pdf]

Supporting Information

This file contains four supplementary figures (Figure S1–Figure S4) and three supplementary tables (Table S1–Table S3) for the metagenomic and metabolomic analysis of a bryozoan-associated microbial complex from the Hanjiang River, China.

Figure S1. Functional annotation of the microbial metagenomes for proteins.

(a) COG functional classification of the microbial metagenomes. Different letters on the diagram correspond to proteins with diverse biological functions; the vertical coordinate indicates the number of unigenes assigned to each functional category in the sample. (b) Abundance of different types of carbohydrate-active enzymes (AA, auxiliary activities; CBM, carbohydrate-binding modules; CE, carbohydrate esterases; GH, glycoside hydrolases; GT, glycosyltransferases; PL, polysaccharide lyases). The vertical axis indicates the number of unigenes encoding each enzyme class.

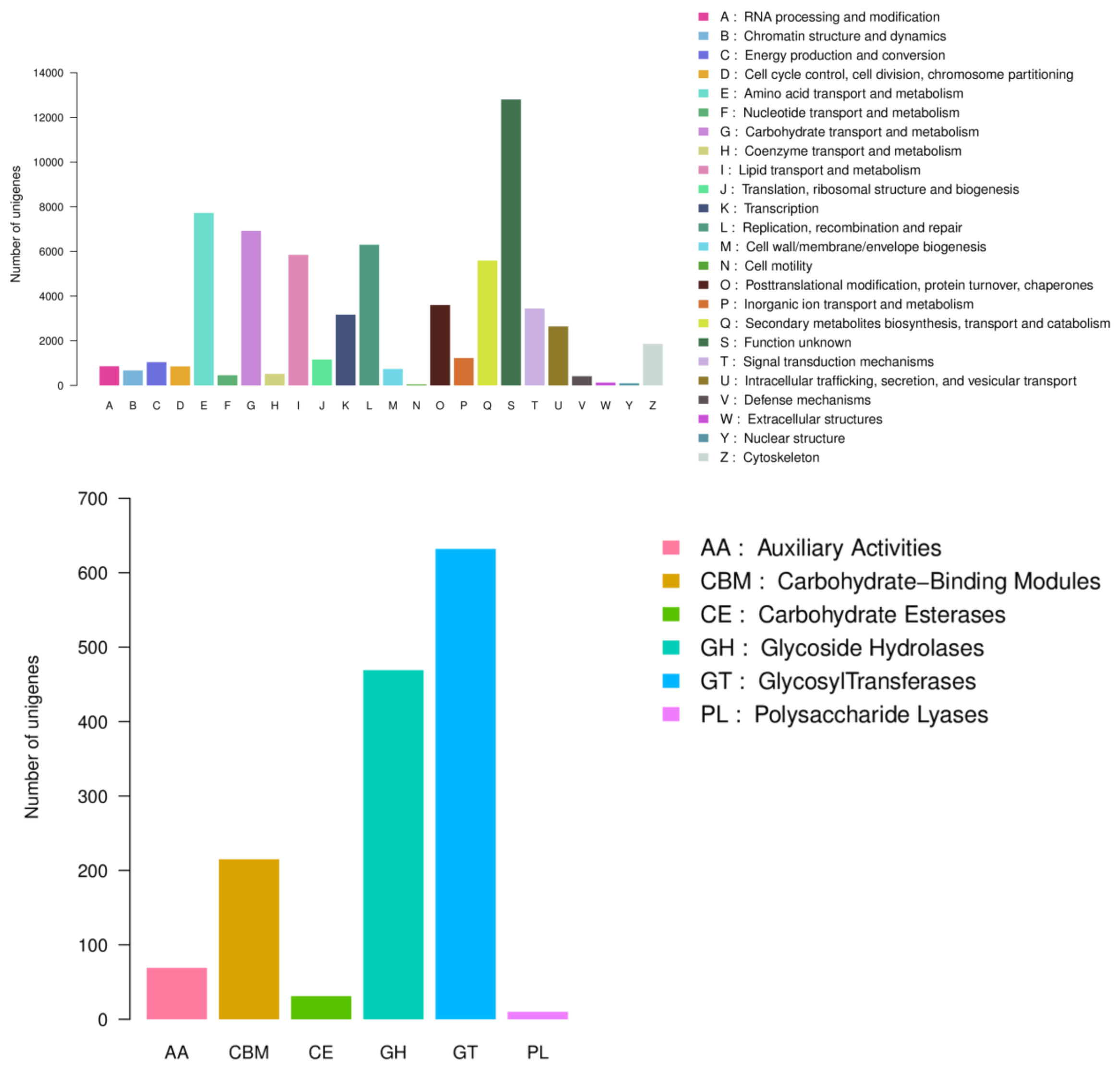

Figure S2. Antibiotic-resistant genes in the microbial complex.

The stacked histogram shows the relative abundance of antibiotic resistance genes in the triplicate samples (MC1, MC2, MC3) of the microbial complex. Different colors indicate different types of resistance genes,

and the area occupied by each colored segment represents the relative abundance of the corresponding resistance gene in the microbial metagenome.

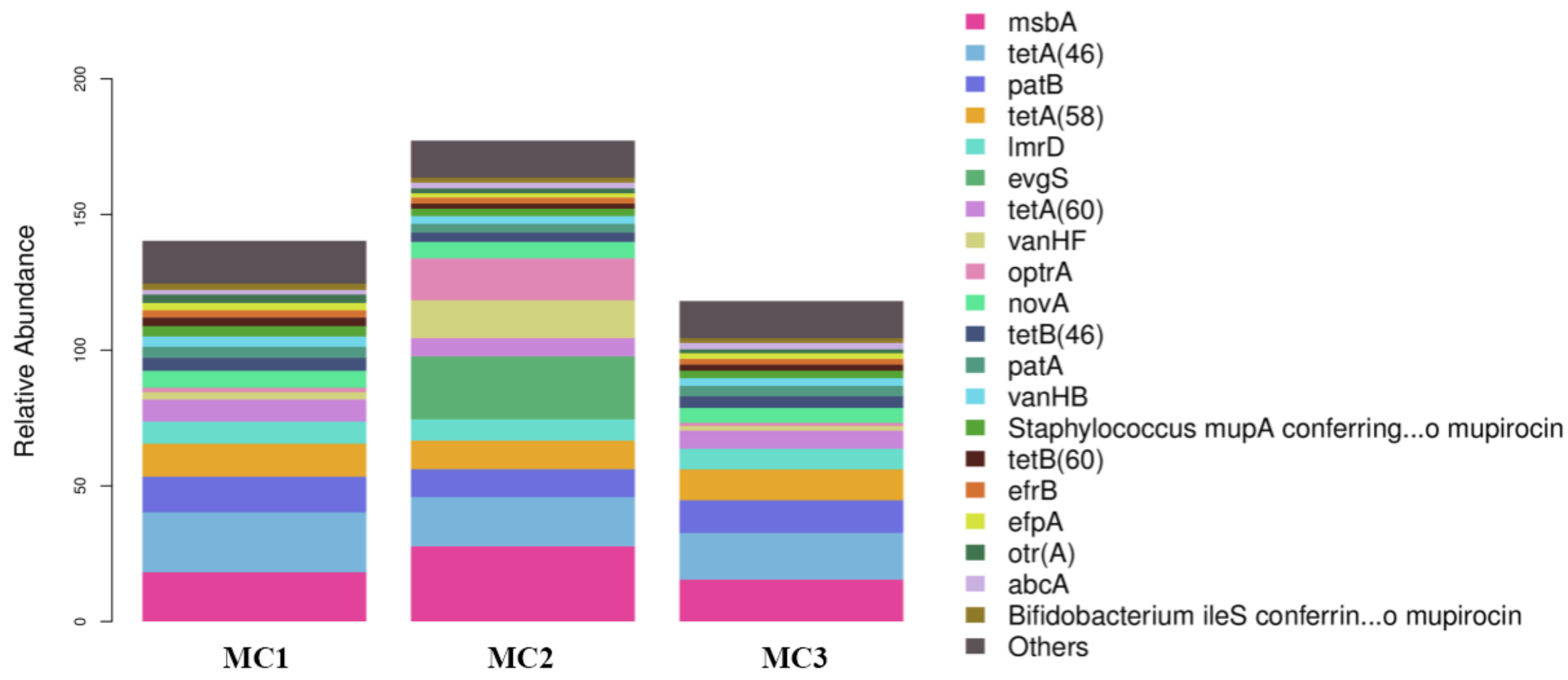

Figure S3. Declined virulence of pathogenic microorganisms found in the bryozoan-dominated microbial complex.

The stacked histogram shows the relative abundance of pathogenic microorganisms in the triplicate samples (MC1, MC2, MC3) according to the type of virulence change (e.g., reduced_virulence, unaffected_pathogenicity, lethal, loss of pathogenicity and their combinations). Different colors indicate pathogenic microorganisms that undergo different virulence changes. The area occupied by each segment indicates the dominance of that change in the overall host–microbe interaction.

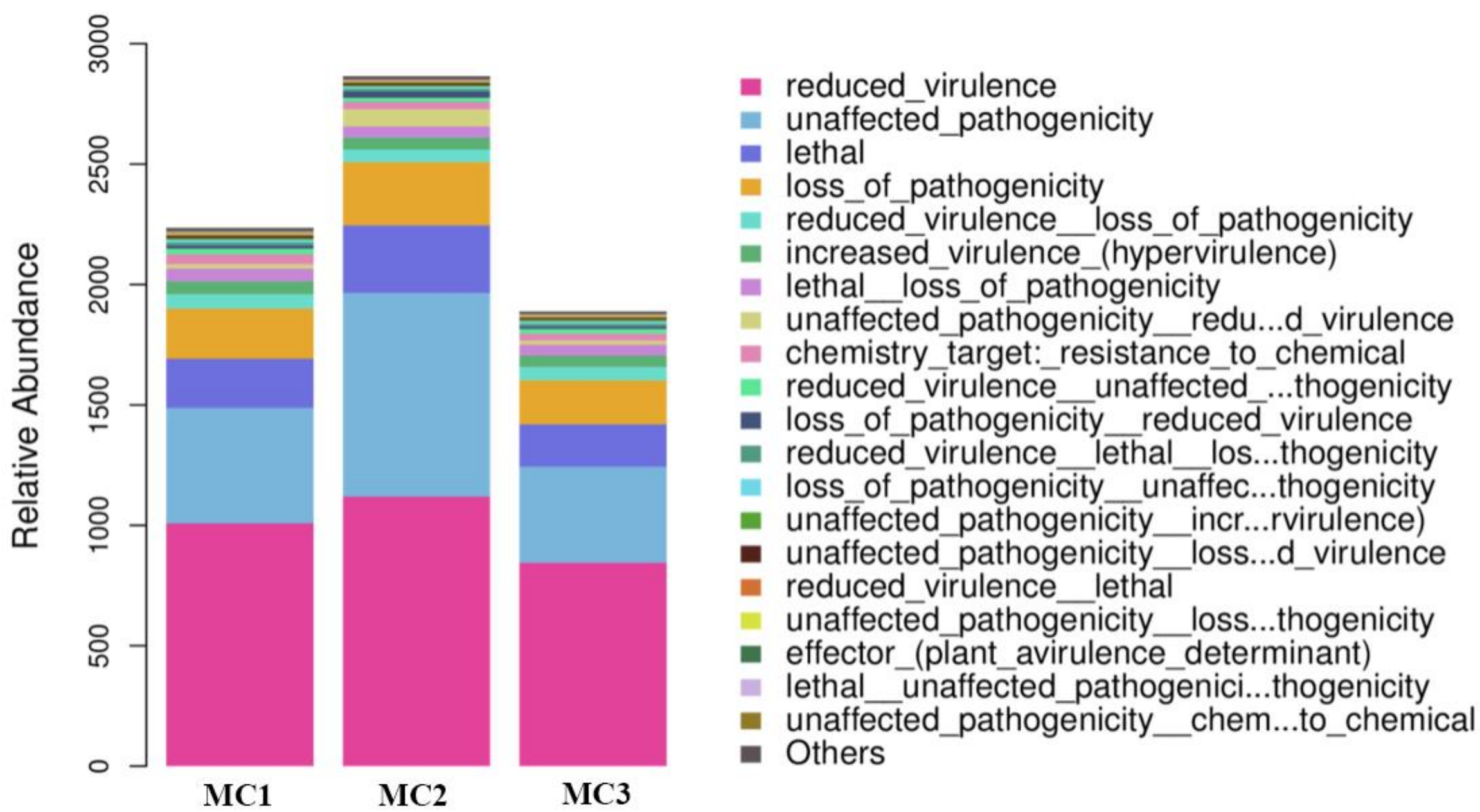

Figure S4. The top 10 metabolites in the microbial complex.

The pie chart shows the ten most abundant primary metabolites of the bryozoan microbial community. Different colors represent different metabolites, and the values indicate the percentage of each metabolite relative to the total metabolite content.

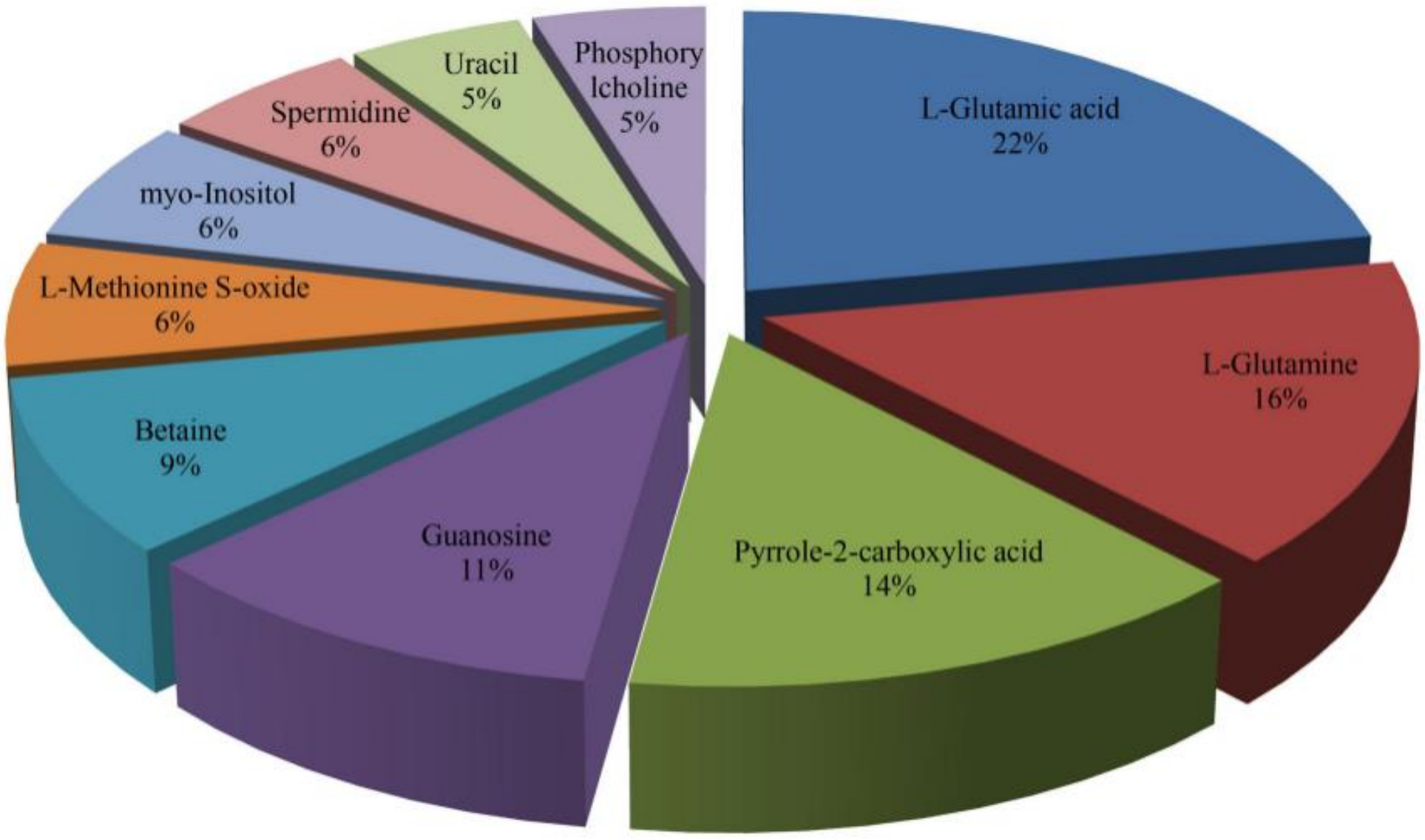

Table S1. Dominant heterotrophic bacteria in the microbial complex.

Category	Number of strains (%)
Pseudomonas	25%
Bacillus	25%
Photobacterium	25%
Acinetobacter	12.5%
Shewanella	12.5%

Table S2. Statistics summary of co-assemblies.

Metric	Average
Total reads	122,519,963
Total bases	18.20 G
Assembly size (bp)	597,490,495
N50 contigs (bp)	5,004
Contigs > 1000 bp	142,948
Longest contig	61,229
Open reading frames	87,383
GC (%)	47.99

Table S3. Type and quantity of metabolites.

ID	Name
M282T279	Guanosine
M191T791	5-Hydroxyindoleacetic acid
M296T438	5-Methylthioadenosine
M375T450	Riboflavin
M89T198	L-Lactic acid
M117T76	Succinic acid
M103T319	(R)-3-Hydroxybutyric acid
M102T81	Dimethylglycine
M116T197	L-Valine
M195T83	Gluconic acid
M122T145	Nicotinic acid
M173T76	trans-Aconitate
M152T701	3-Hydroxyanthranilate
M121T500	Phenylethylamine

ID	Name
M249T676_1	gamma-Glutamylcysteine
M151T412	p-Anisic acid
M218T151	Pantothenic acid
M147T672_1	trans-Cinnamate
M163T151	4-Hydroxycinnamic acid
M227T567	Prephenate
M352T162	Adenosine 2,3-cyclic phosphate
M268T153	Adenosine
M227T400	L-Arogenate
M167T799	Perillic acid
M135T811	Perillyl alcohol
M215T109	Dethiobiotin
M171T590	1-Hydroxy-2-naphthoate
M211T733	(+)-7-Isojasmonic acid
M325T93	Sucrose
M200T688	Dodecanoic acid
M319T790	5-KETE
M321T814	14,15-DiHETrE
M140T565	Acetylphosphate
M171T490	Dihydroxyacetone phosphate
M90T664	Putrescine
M113T143	Uracil
M112T142	Cytosine
M102T663	Succinic acid semialdehyde
M133T84	L-Asparagine
M88T513	Butyric acid
M147T86	L-Glutamine
M118T90	Betaine
M166T90	L-Methionine S-oxide
M132T547	N-Carbamoylputrescine
M184T86	Phosphorylcholine
M136T103	Adenine
M111T801	Pyrrole-2-carboxylic acid
M127T317	Thymine
M159T243	4,5-Dihydroorotic acid
M132T114_1	Glutaric acid
M116T149	L-Proline
M132T680	cis-4-Hydroxy-D-proline
M132T280	5-Aminolevulinic acid
M148T89	L-Glutamic acid
M131T742	Ketoleucine
M146T587	2-Dehydropantoate
M164T738	L-Rhamnofuranose
M181T802	myo-Inositol
M132T478	L-Isoleucine
M132T289	Aminocaproic acid
M182T549	Sorbitol
M139T104	Urocanic acid
M111T674	Hydroquinone
M127T403	Maltol
M127T279	1,2,3-Trihydroxybenzene
M94T70	Aniline
M143T649	5-(2-Hydroxyethyl)-4-methylthiazole
M145T730	Spermidine

ID	Name
M122T155	3-Hydroxybenzaldehyde
M109T543	m-Cresol
M124T793	3-Hydroxybenzyl alcohol glucoside
M172T403	3-Dehydroshikimate
M170T373	Norepinephrine
M200T311	gamma-Glutamyl-beta-aminopropiononitrile
M153T680	3-Hydroxyphenylacetic acid
M169T70	(S)-4-Hydroxymandelate
M184T363	3,4-Dihydroxymandelic acid
M167T73	L-4-Hydroxyphenylglycine
M166T150	D-Phenyllactic acid
M228T794	Deoxyuridine
M132T401	3-Methylindole
